# Liprin-α1 is a regulator of vimentin intermediate filament network in the cancer cell adhesion machinery

**DOI:** 10.1038/srep24486

**Published:** 2016-04-14

**Authors:** Henna Pehkonen, Pernilla von Nandelstadh, Piia-Riitta Karhemo, Tatiana Lepikhova, Reidar Grenman, Kaisa Lehti, Outi Monni

**Affiliations:** 1 Research Programs Unit, Genome-Scale Biology Research Program and Institute of Biomedicine, Medical Biochemistry and Developmental Biology, 00014 University of Helsinki, Finland; 2 Department of Otorhinolaryngology, Head and Neck Surgery, Turku University and Turku University Hospital, Finland; 3 Department of Microbiology, Tumor and Cell Biology, Karolinska Institutet, Stockholm, Sweden; 4 K. Albin Johansson Senior Researcher, Finnish Cancer Institute, Finland

## Abstract

*PPFIA1* is located at the 11q13 region, which is one of the most commonly amplified regions in several epithelial cancers including head and neck squamous cell carcinoma and breast carcinoma. Considering the location of *PPFIA1* in this amplicon, we examined whether protein encoded by *PPFIA1*, liprin-α1, possesses oncogenic properties in relevant carcinoma cell lines. Our results indicate that liprin-α1 localizes to different adhesion and cytoskeletal structures to regulate vimentin intermediate filament network, thereby altering the invasion and growth properties of the cancer cells. In non-invasive cells liprin-α1 promotes expansive growth behavior with limited invasive capacity, whereas in invasive cells liprin-α1 has significant impact on mesenchymal cancer cell invasion in three-dimensional collagen. Current results identify liprin-α1 as a novel regulator of the tumor cell intermediate filaments with differential oncogenic properties in actively proliferating or motile cells.

Liprin-α1 is encoded by *PPFIA1* located at the 11q13 locus, which is one of the most commonly amplified region in several epithelial cancers such as those arising in head and neck^1^,^2^, breast^3^,^4^, and ovarian cancer^5^,^6^. The importance of 11q13 amplification in epithelial cancers is highlighted by its association with poor prognosis^3^,^7^,^8^. In squamous cell carcinoma of head and neck, *PPFIA1* shows high correlation between DNA copy number and gene expression^1^,^2^,^7^,^9^. Despite its location in commonly amplified region, the role of liprin-α1 in cancer progression has not been studied in detail. Furthermore, the previously reported function of liprin-α1 for epithelial cancer cell migration is controversial. In head and neck cancer cells, depletion of liprin-α1 enhances migratory properties^10^, whereas in breast cancer cells its depletion leads to decreased migration and extracellular matrix (ECM) degradation^11^. In colon carcinoma cells, liprin-α1 has a positive effect on cell motility, which has been linked to interaction with the tumor suppressor protein *ING4*^12^.

Liprin-α1 belongs to the liprin (LAR[leukocyte common antigen related]-interacting protein) family, which consists of liprin α- and β- cytosolic adaptor and scaffold proteins^13^. Liprin-α proteins have been mainly studied in neurons, where they function in active synaptic zones and post-synaptic sites^14^,^15^ as well as have a role in synaptic vesicle trafficking^16^,^17^. In non-neuronal cells, liprin-α proteins have several functions related to cell adhesion, signal transduction and organization of the cytoskeleton^18^,^19^,^20^. Importantly, liprin-α1 is essential for the regulation of invadosome function and cell spreading as well as focal adhesion dynamics^11^,^19^. Regulation of focal adhesion dynamics is a complex process that involves interplay of various proteins, including those at the actin cytoskeleton and vimentin intermediate filament networks, which are interlinked in an adhesion dependent manner^21^. Vimentin network regulates focal adhesion size and focal contacts, functioning as both the scaffold and stabilizer for cell-matrix adhesion^22^,^23^.

Besides focal adhesions, podosomes, which are dynamic multiprotein structures with an outer adhesion ring and a cytoskeletal core, contribute to cell adhesion. Podosomes are found widely in motile cells such as macrophages, endothelial cells, transformed fibroblasts, osteoclasts, malignant B lymphocytes and carcinoma cells^24^,^25^,^26^,^27^,^28^,^29^,^30^. Podosomes can degrade ECM, they stabilize cellular elements, and are linked to directional movement^31^,^32^,^33^. In cancer cells podosome-like adhesion structures with adhesion rings, are proposed to be required for maturation of invadopodia^34^. In addition, podosome-like structures have been proposed as precursors for invadopodia, the ECM degrading structures that promote invasion of the transformed cells^31^,^35^,^36^,^37^. Due to many similar characteristics, the terms podosomes and invadopodia are sometimes used interchangeably. To avoid misunderstanding, a term invadosome was recently introduced to describe all the adhesive structures that are involved in ECM degradation and invasion^38^.

The aim of our study was to investigate the potential role of liprin-α1 in cellular signaling related to the adhesion, migration and invasion of cells from squamous cell carcinoma of head and neck and breast cancer. We discovered that liprin-α1 localizes to the distinct cell adhesion structures, and regulates the intermediate filament vimentin network, thereby exerting effects on cell invasive growth.

## Results

### Liprin-α1 regulates tumor cell invasive growth in 3D collagen

**Figure 1:**
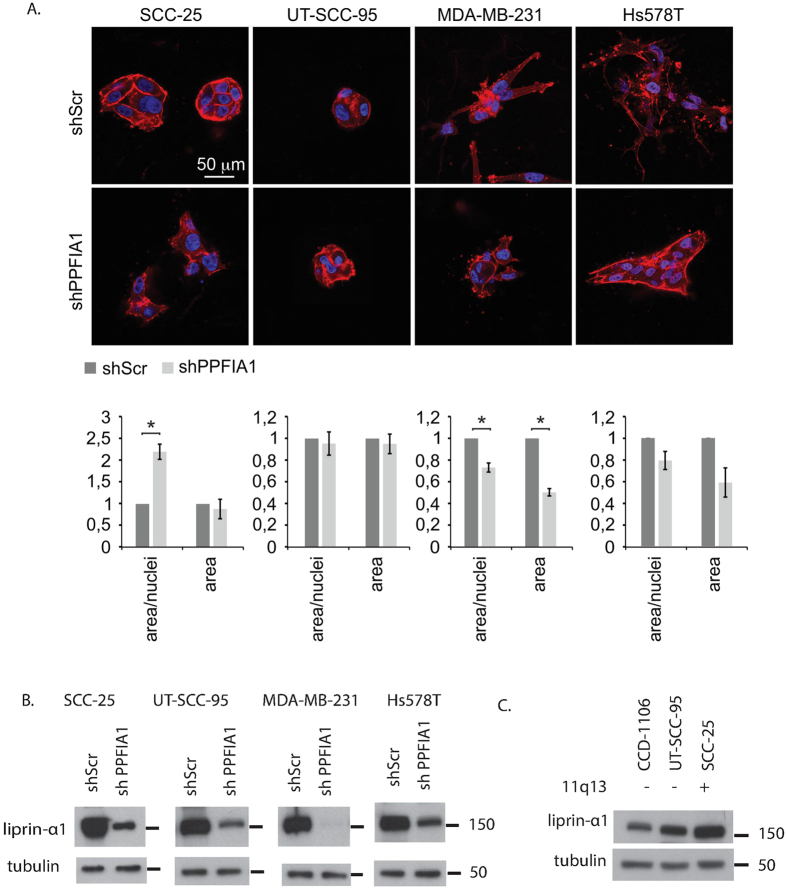
Liprin-α1 regulates cell invasive growth in collagen. (**A**) HNSCC and breast carcinoma cells were embedded in 3D collagen after liprin-α1 silencing and cultured for 5 days. Confocal micrographs show F-actin (phalloidin, red) and nuclei (DAPI, blue) in representative colonies. Quantification of the invasive growth is expressed as relative area of colonies or relative area/nuclei; mean ± SEM; three collagen preparations/stable control (shScr) or knockdown (shPPFIA1) cell line. *P < 0.05, unpaired Student’s *t*-test. (**B**) Western blots verifying liprin-α1 knockdown in SCC-25, UT-SCC-95, MDA-MB-231 and Hs578T cell lines. (**C**) HNSCC cells with *PPFIA1* amplification show higher expression of liprin-α1.

Contradictory results have been reported regarding the effects of liprin-α1 on the motility of head and neck squamous cell carcinoma (HNSCC) and breast cancer cell lines (Table 1)^10^,^11^, although both these cell types display frequent 11q13 amplification. Therefore, to examine the potential functions of liprin-α1 in migration and invasive growth of HNSCC and breast carcinoma cells, UT-SCC-95, SCC-25 and MDA-MB-231 cells were subjected to transwell migration assays through Matrigel (Supplementary Figure 1A). Because HNSCC cells barely migrated through this matrix we embedded the cells as single-cell suspension in three-dimensional (3D) collagen that typifies the tumor adjacent ECM. During a 5-day culture, the control SCC-25 and UT-SCC-95 cells (shScr) displayed an expansive growth behavior with limited invasive activity into the cell-surrounding collagen. In SCC-25 cells, the knockdown of endogenous liprin-α1 by lentiviral shRNA diminished the cell-cell adhesive growth pattern and turned the cells to a more invasive phenotype with less prominent cell-cell contacts in 3D collagen. Liprin-α1 knockdown did not significantly affect UT-SCC-95 cell invasion, although less regular colonies and actin assembly to cell edges and protrusions was observed also in these cells (Fig. 1A). In the highly invasive MDA-MB-231 and Hs578T breast cancer cells that display different types of multi-cellular outgrowths and singly invading cells with mesenchymal morphology, liprin-α1 knockdown instead reduced the colony size and invasion (Fig. 1A). Efficiency of liprin-α1 knockdown was verified by western blot (Fig. 1B), and liprin-α1 protein levels were confirmed to correlate with status of *PPFIA1* amplification in HNSCC cell lines (Fig. 1C). Our data suggest that, liprin-α1 is needed for the expansive growth behavior and prominent intercellular contacts of the primary HNSCC cells within 3D collagen, whereas in MDA-MB-231 and Hs578T cells liprin-α1 promotes mesenchymal cell invasion, concurring with the previous results of opposite liprin-α1 effects reported in HNSCC and breast cancer cell invasion.

### Liprin-α1 localizes to adhesion rings in HNSCC from primary tumor

**Figure 2:**
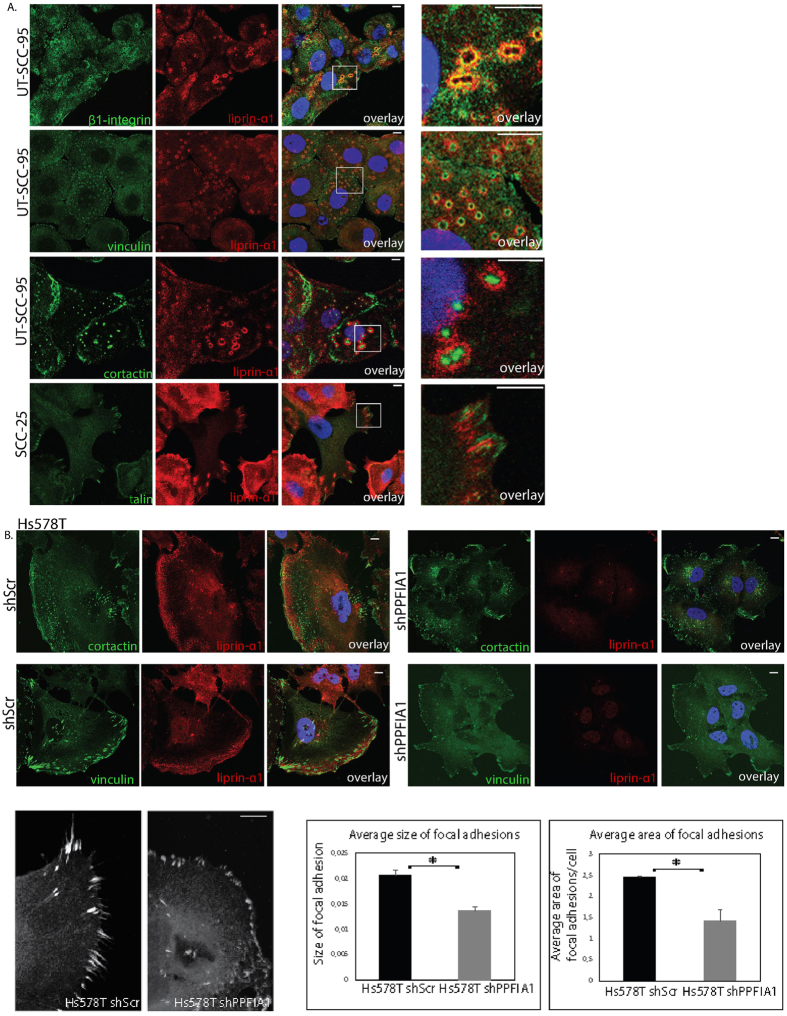
Localization of liprin-α1 and other adhesion proteins in HNSCC cell lines from primary tumor and effect of liprin-α1 knockdown on cortactin and vinculin expression in invasive breast cancer cells. (**A**) Co-localization of liprin-α1 with activated β1 integrins in adhesion rings and localization of vinculin and cortactin into the inner part and core of adhesion rings, respectively, in UT-SCC-95 HNSCC cells. Localization of talin and liprin-α1 in adhesion structures in SCC-25 HNSCC cell line. The last figure in each panel shows the enlargement of the overlay image, as indicated by the scale bar. (**B**) Knockdown of liprin-α1 did not inhibit localization of cortactin in invadopodia as compared to the control cells transduced with scrambled shRNA in Hs578T breast cancer cells. Vinculin staining reveals differences in the shape and size of focal adhesions in Hs578T breast cancer cells after liprin-α1 knockdown as compared to control cells. The scale bar is 10 μm. *P < 0.05, unpaired Student’s *t*-test.

In order to examine the potential direct liprin-α1 functions in cell-cell adhesion, we followed spheroid formation of the control (shScr) and liprin-α1 knockdown (shPPFIA1) HNSCC cells in low-adherent plates in the absence of the 3D matrix. However, the spheroid formation and cell-cell contacts displayed by the control UT-SCC-95 and SCC-25 cells remained unaltered after liprin-α1 knockdown (Supplementary Figure 1B). This raised the possibility that, instead of direct regulation of intercellular junctions, liprin-α1 alters cell-cell contacts and invasion via collagen and β1-integrin mediated signals. To study whether liprin-α1 is involved in β1-integrin signalling and adhesion or cytoskeletal structures, the localization of liprin-α1, other focal adhesion proteins, and the 11q13 encoded protein cortactin were analyzed by immunofluorescence in HNSCC cell lines from primary tumor from tongue. In the UT-SCC-95 cells, liprin-α1 was prominently localized to the adhesion rings, where it co-localized with activated β1-integrin (Fig. 2A), whereas vinculin was located closer to the cortactin- and actin-rich cores of the adhesive structures (Fig. 2A; Supplementary Figure 2A). Instead, in the SCC-25 HNSCC cell line, liprin-α1 localized primarily near or at the focal adhesions and less prominently to adhesion rings (Fig. 2A, Supplementary Figure 2B). In general, liprin-α1 displayed a more distant localization at the outer edges of the adhesion rings compared to pFAK, paxillin and talin (Supplementary Figure 2A,B). Interestingly, endogenous liprin-α1 and talin both localized in the same adhesive structures (Fig. 2A, Supplementary Figure 2B) and liprin-α1 co-immunoprecipitated with talin in SCC-25 and UT-SCC-95 cell lines (Supplementary Figure 2B).

Since liprin-α1 had contribution to invasive cell growth of breast cancer cells into collagen I (Fig. 1), we compared localization of liprin-α1 in HNSCC and in invasive breast cancer cell lines. In Hs578T and MDA-MB-231 breast cancer cells, liprin-α1 was detected near or at the lamellipodia (Fig. 2B, Supplementary Figure 3A) suggesting that this localization is typical for migrating cells that lack strong adhesion rings. In our study, liprin-α1 and cortactin were not found in the same structures of the breast cancer cell lines because these cells did not form adhesion rings as compared to HNSCC cells originating from primary tumor (Fig. 2). Knockdown of liprin-α1 changed the focal adhesion morphology to smaller adhesions at the periphery of Hs578T breast cancer cells (Fig. 2B).

### Liprin-α1 is involved in extracellular matrix degradation in HNSCC and localizes to different adhesion related structures depending on cell origin

**Figure 3:**
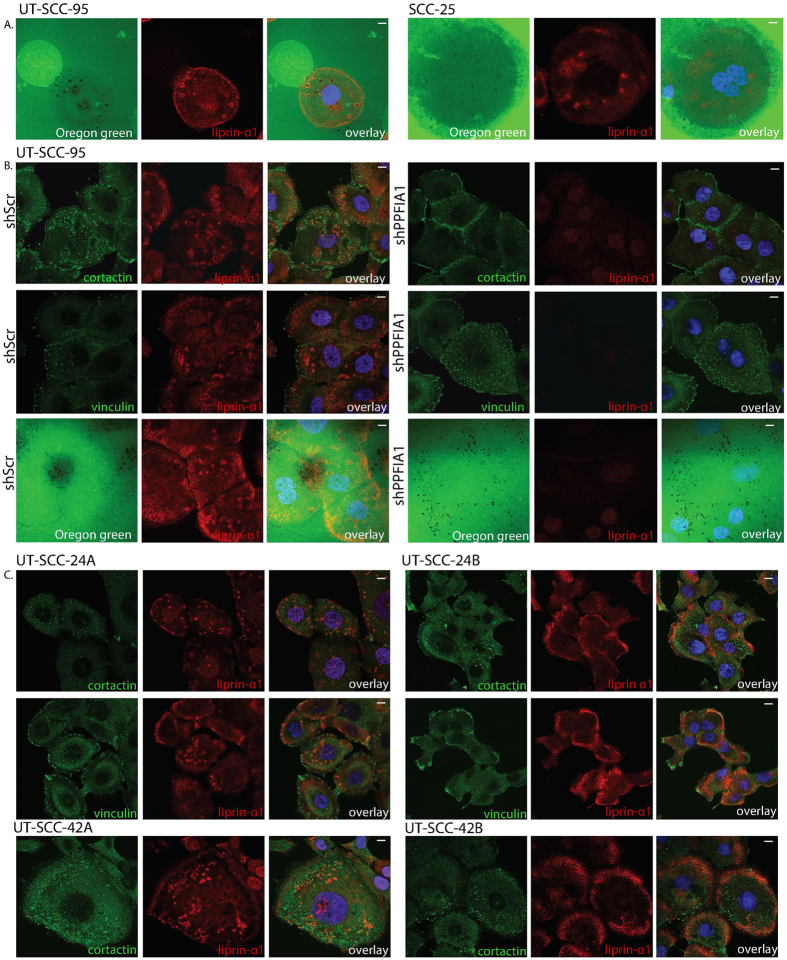
Liprin-α1 localizes to extracellular matrix degrading complexes and liprin-α1 localization differs based on the origin of the cell line. (**A**) Localization of liprin-α1 at the extracellular degrading complexes in HNSCC. Degrading areas are shown in black and fluorescent gelatin in green. (**B**) Knockdown of liprin-α1 did not prevent adhesion ring formation or ECM degradation in non-invasive HNSCC. (**C**) Liprin-α1 localization differs between the cell lines obtained from primary tumor and metastasis from the same patient. Liprin-α1 localized to invadosome adhesion rings with cortactin and vinculin in HNSCC cell line from primary tumor (UT-SCC-24 A and UT-SCC-42 A), whereas in metastatic cell lines (UT-SCC-24B and UT-SCC42B) liprin-α1 was located near cell edge and cortactin localized to invadopodia. The scale bar is 10 μm.

To determine the potential function of liprin-α1 related to invadosome activity, we first analyzed the ability of primary HNSCC cells to degrade ECM. The gelatin degradation assay revealed that liprin-α1 containing adhesion rings in the UT-SCC-95 and SCC-25 cell lines were able to degrade ECM (Fig. 3A). To analyze, if liprin-α1 regulates adhesion ring mediated matrix degradation, we used lentiviral shRNA to knockdown liprin-α1 in the UT-SCC-95 cell line. However, cortactin, vinculin and actin were recruited to the adhesion rings after liprin-α1 knockdown, indicating that liprin-α1 was not essential for adhesion ring formation (Fig. 3B). Liprin-α1 knockdown neither prevented the matrix degrading capability of the UT-SCC-95 cells (Fig. 3B, Supplementary Figure 3B). Our results suggest, that in contrast to the previously reported functions in the dynamics of ECM degradation and invadosomes in the invasive MDA-MB-231 cells^11^, depletion of liprin-α1 does not affect the capability of poorly collagen-invasive cells to degrade extracellular matrix. Strong adhesion ring formation in non-invasive cells can explain the differences between cell lines.

Due to the fact that liprin-α1 knockdown affected invasive cell growth differently in primary HNSCC and metastatic breast cancer cells, we compared the localization of liprin-α1 in primary and primary persistent or metastatic cell lines from the same HNSCC patient. Similar to other HNSCC cell lines from primary tumor, liprin-α1 was found either around the cortactin-containing core outside of vinculin or at the lamellipodia at the vicinity of vinculin positive small focal adhesions (Fig. 3C). Adhesion ring formation was not specific for one cell line, but occurred in several head and neck cancer cell lines derived from primary tumors (Figs 2A and 3C). Instead liprin-α1 was mostly located near or at the lamellipodia together with vinculin in corresponding metastatic cell lines (Fig. 3C). This finding is in line with the localization of liprin- α1 in metastatic breast cancer cells (Fig. 2B). Our experiments also showed that in metastatic cells with large and spread lamellipodia, liprin-α1 was detected in a front near lamellipodia, as well as at the leading edge partially localizing with vinculin-positive focal adhesions (Supplementary Figure 3C). As observed in breast cancer cells (Fig. 2B), knockdown of liprin-α1 did not prevent formation of focal adhesions or cortactin-positive structures in metastatic or primary persistent HNSCC cell lines, although focal adhesions were smaller or more elongated than in control cells (Supplementary Figure 4A,B).

**Figure 4:**
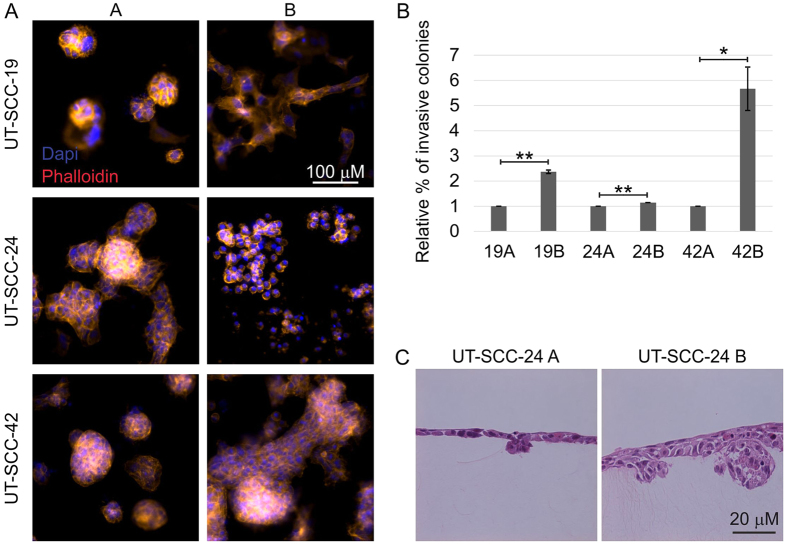
Invasive growth of primary (**A**) and metastatic or primary persistent cell lines (**B**) from the same patient. (**A**) HNSCC carcinoma cells were embedded in 3D collagen as a single cell suspension. Representative confocal micrographs show F-actin (phalloidin) and nuclei (DAPI) in cell colonies after 8 days in culture. (**B**) Quantification of the invasive growth is expressed as relative percentage of invading colonies; the A cell line was set as 1. The error bars indicate mean ± SEM. Five collagen preparations/cell line. *P < 0.01, **P < 0.001, unpaired Student’s *t*-test. (**C**) Light micrographs of collagen cross sections visualize H&E-stained cells after 9 days in culture.

Due to differences in the role of liprin-α1 in promoting invasion in primary and invasive cancer cells, we examined the invasion capabilities of primary and metastatic HNSCC cell lines obtained from the same patient. Our results showed that metastatic or primary persistent HNSCC cancer cells (UT-SCC-19B, −24B and −42B) displayed higher invasive capacity than corresponding cells from primary tumor (UT-SCC-19A,−24A and −42A) (Fig. 4) explaining differences in localization of liprin-α1 and cortactin in different types of cell lines. In metastatic HNSCC cell lines liprin-α1 showed same localization pattern as compared to invasive breast cancer cells (Figs 2 and 3).

### Liprin-α1 regulates intermediate filament vimentin

To study the more precise mechanism of liprin-α1 function, we assessed the effect of liprin-α1 knockdown or overexpression to gene expression by microarray analysis. Interestingly, liprin-α1 knockdown altered the gene expression levels of *VIM* (Table 2). We also found, that *PPFIA1* alters expression of *MMP13* (Table 2) located at the 11q22 region which is reported to be related to cell invasion, growth and aggressiveness of HNSCC^39^,^40^,^41^. Moreover, while the intermediate filament keratins *KRT1, KRT4*, *KRT10* and *KRT13* were downregulated, *VIM* was upregulated in *PPFIA1* shRNA treated cells (Table 2). In addition, liprin-α1 overexpression in UT-SCC-95 cells reduced the *VIM* expression and upregulated *MMP13* (Table 3).

**Figure 5:**
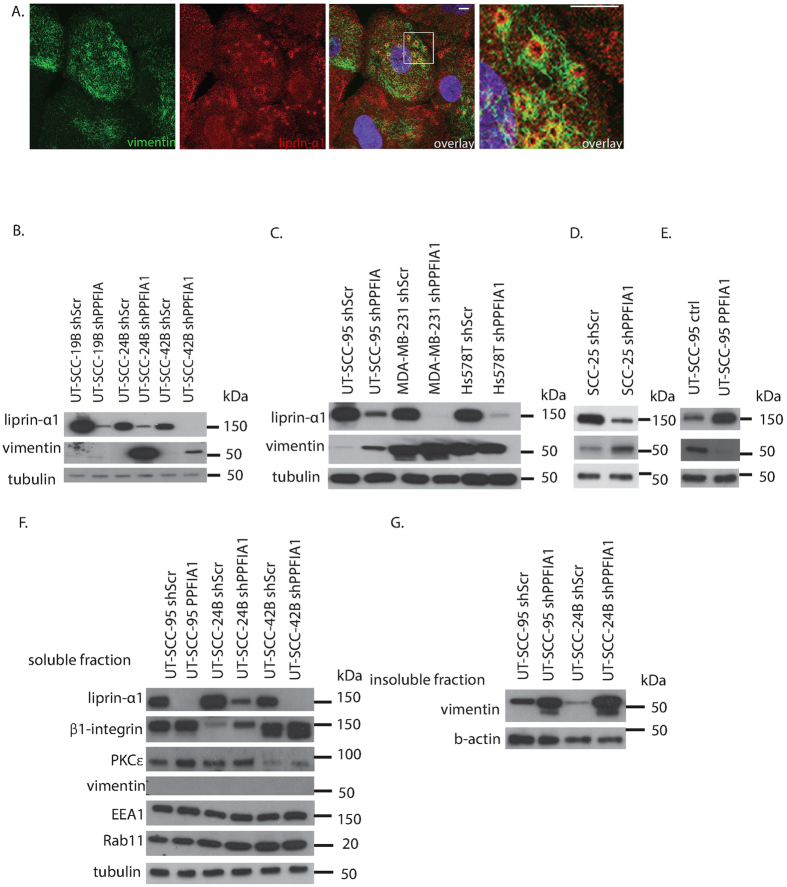
Knockdown of liprin-α1 affects vimentin expression in HNSCC cells. (**A**) Localization of vimentin intermediate filaments around and close to adhesion rings in UT-SCC-95 HNSCC cell line. The last figure in the panel shows the enlargement of the overlay, as indicated by the scale bar. Scale bar is 10 μm. (**B**) Effect of liprin-α1 knockdown on vimentin expression in metastatic and primary persistent HNSCC cell lines (**C**,**D**) Effect of liprin-α1 knockdown on vimentin expression in UT-SCC-95 and SCC-25 HNSCC cell lines and highly invasive breast cancer cell lines MDA-MB-231 and Hs578T. (**E**) Vimentin expression was decreased upon liprin-α1 overexpression in UT-SCC-95 cell line. (**F**) Knockdown of liprin-α1 led to accumulation of total β-integrin in metastatic cell lines and slight increase in PKCε expression. Knockdown of liprin-α1 did not affect the expression of EEA1 (early endosomes) or Rab11 (recycling endosomes) in soluble fraction of HNSCC cells. (**G**) Liprin-α1 knockdown caused vimentin accumulation in insoluble fraction of HNSCC cell lines.

In the UT-SCC-95 cell line vimentin intermediate filaments were localizing with liprin-α1 around adhesion ring (Fig. 5A). Vimentin upregulation after knockdown of liprin-α1 was observed in several HNSCC cell lines. Upregulation was detected in metastatic UT-SCC-24B and UT-SCC-42B cell lines (Fig. 5B) as well as in primary UT-SCC-95 and SCC-25 HNSCC cells (Fig. 5C,D). Vimentin expression was significantly lower in control HNSCC cells as compared to breast cancer cells, which express high levels of vimentin (Fig. 5C). In the UT-SCC-95 cell line, overexpression of liprin-α1 led to vimentin downregulation, further supporting our results on a regulative role of liprin-α1 in vimentin expression (Fig. 5E). Increased vimentin expression was only detected in the insoluble or cytoskeletal fraction of knockdown cells (Fig. 5F,G). Both vimentin and liprin-α1 has been shown to regulate β1-integrin trafficking^42^,^43^. Therefore, we wanted to test whether liprin-α1 knockdown has any effect on β1-integrin levels in HNSCC cells. β1-integrin was accumulating in the soluble fraction of the cells upon liprin-α1 knockdown, but no effect was found on the early (EEA1) or recycling (Rab11) endosome proteins (Fig. 5F). On the other hand PKCε, which is necessary for integrin recycling, was slightly upregulated together with β1 integrins in the soluble fraction of knockdown cells (Fig. 5F). Our data supports previous findings showing that liprin-α1 participates in β1-integrin signalling, required for effective migration, particularly in motile cells.

**Figure 6:**
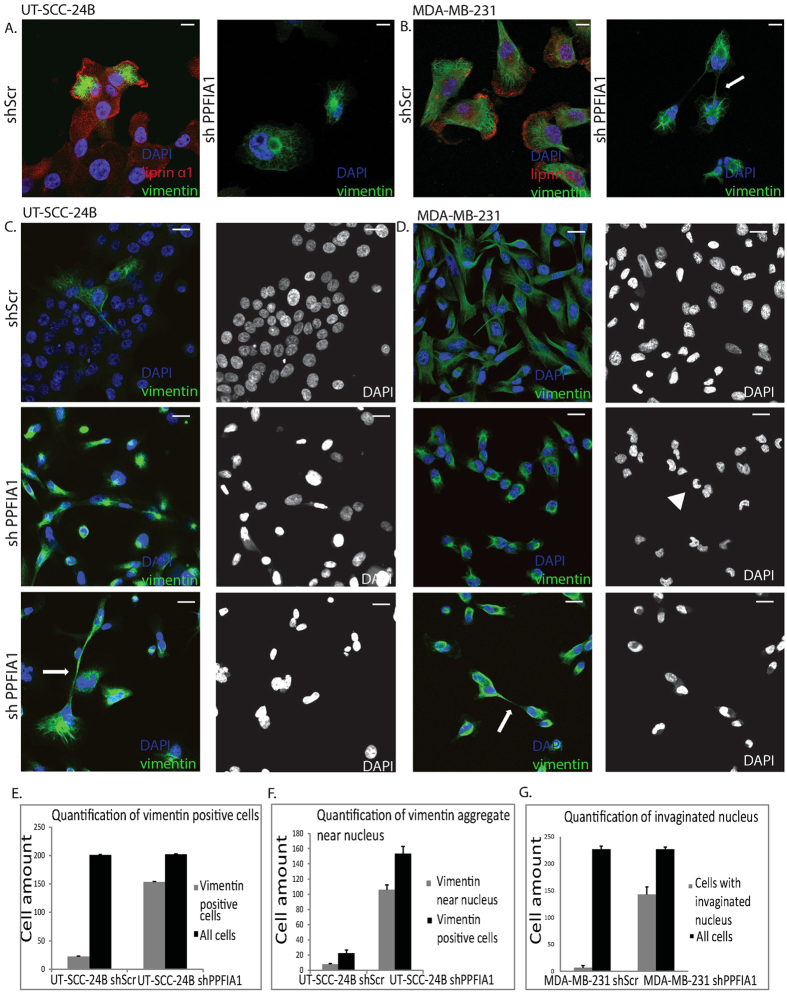
Liprin-α1 knockdown causes changes in vimentin intermediate filament network in HNSCC and breast carcinoma cells. (**A**,**B**) Liprin-α1 and vimentin staining in control cells and vimentin structures near nucleus or vimentin bridge in metastatic HNSCC and breast cancer cells with knockdown of liprin-α1. The scale bar is 10 μm. (**C**,**D**) Formation of vimentin bridge structures as well as multinucleated and irregular nuclei in metastatic HNSCC and breast cancer cells with knockdown of liprin-α1. (**E**) Quantification of vimentin positive cells in UT-SCC-24B cells with shScr and shPPFIA1 liprin-α1 knockdown. (**F**) Quantification of vimentin aggregates located near nucleus in shScr and shPPFIA1 liprin-α1 knockdown UT-SCC-24B cells. (**G**) Quantification of invaginated nuclei in shScr and shPPFIA1 knockdown cells in the MDA-MB-231 cell line. Error bars indicate standard deviations (s.d.) from three independent experiments. P < 0.05, unpaired Student’s *t*-test. Arrow head indicates invaginated nucleus. Arrows indicate vimentin bridges. The scale bar is 20 μm.

Vimentin was highly expressed at the cell colony edge in UT-SCC-24B cells, whereas MDA-MB-231 showed a strong vimentin network throughout the cells (Fig. 6A,B). Liprin-α1 knockdown led to vimentin accumulation near the nucleus in both of these cells (Fig. 6A,B) as well as formation of vimentin bridges and multinucleated cells in both the HNSCC and breast cancer cell lines (Fig. 6). Furthermore, invaginated nuclei were found in breast cancer cells (Fig. 6D,G). Our data suggests that liprin-α1 stabilizes the cell membrane and cytoskeletal elements in motile cells. Although knockdown of liprin-α1 caused an effect on vimentin expression in several cell lines, it did not alter significantly the levels of other proteins related to epithelial to mesenchymal transition or other focal adhesion proteins nor cortactin (Supplementary Figure 5A–C). Similarly, knockdown of cortactin did not significantly affect the levels of any of the studied focal adhesion proteins or liprin-α1 (Supplementary Figure 5C). As an important conclusion, our data shows for the first time that liprin-α1 regulates the vimentin intermediate filament network influencing cell division and morphology.

### Liprin-α1 knockdown affects cell growth in several cancer cell lines

**Figure 7:**
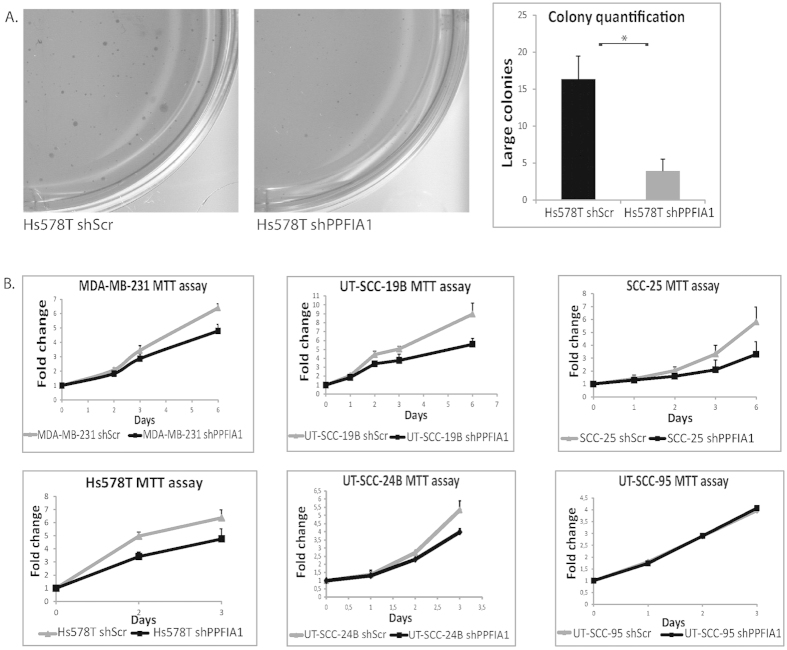
Effect of liprin-α1 knockdown on cell growth. (**A**) Liprin-α1 knockdown reduces the size of colonies in Hs578T breast cancer cells grown in soft agar. Error bar was calculated from three independent experiments and indicate standard deviations (s.d.). *P < 0.05, unpaired Student’s *t*-test. (**B**) MTT- proliferation/cell viability assays for different HNSCC and breast cancer cell lines. Each experiment was done in triplicate. The difference in cell growth between *PPFIA1* shRNA cells and Scr shRNA cells was statistically significant at the last day of the experiment (P < 0.05, unpaired Student’s *t*-test, error bars indicate s.d. calculated from three independent experiments) in all cell lines except for UT-SCC-95.

Cytoskeletal reorganization as well as changes in focal adhesion dynamics are related to cell transformation during cancer progression^44^. Because liprin-α1 knockdown led to formation of vimentin accumulation and vimentin bridges (Fig. 6), we next assessed the effect of liprin-α1 to anchorage independent cell growth in soft agar. HNSCC cell lines showed only limited growth in soft agar (data not shown), whereas the invasive breast cancer cells, which had undergone epithelial to mesenchymal transition showed colony forming capability (Fig. 7A). Breast cancer cells were used to assess the effect of liprin-α1 on cell growth in highly motile and invasive cells, with less cell-cell contacts. Liprin-α1 knockdown reduced the size of colonies in soft agar (Fig. 7A). MTT-assay for cell growth showed reduced metabolic activity in liprin-α1 knockdown cells compared to control cells when breast cancer and HNSCC cell lines were grown in 2D culture (Fig. 7B). However, when liprin-α1 was knocked down in the UT-SCC-95 cell line, which does not have 11q13 amplification and has lower expression level of liprin-α1 than those with amplification, there was no effect on cell growth. This implies that the function of liprin-α1 in promoting oncogenic properties is emphasized in more motile and actively proliferating cells.

## Discussion

Liprin-α1 is a member of the LAR protein-tyrosine phosphatase-interacting protein family with a role in cell edge dynamics and cell motility^18^,^19^,^43^. Liprin-α1 is encoded by *PPFIA1*, which is located at the 11q13 region. *PPFIA1* maps next to *CTTN*, which encodes for cortactin, and they are co-amplified in 20–30% of HNSCC^1^,^2^ and 10–20% of breast cancer cells^45^. Despite the location of *PPFIA1* in one of the most commonly amplified regions in many epithelial cancers, the role of liprin-α1 in cancer progression has not been studied precisely. Previous studies show contradictory role of liprin-α1 in cancer cell invasion. Liprin-α1 depletion increases invasion of head and neck squamous cell carcinoma cells^10^, whereas the effect is opposite on breast cancer^11^. Our aim herein was to study the function of liprin-α1 and evaluate whether *PPFIA1*/liprin-α1 possesses oncogenic properties in cancers where it is often amplified. Our results support the recent findings in breast cancer, because knockdown of liprin-α1 reduced the colony size and cell invasive growth in 3D collagen for highly invasive breast cancer cells. On the other hand, in HNSCC UT-SCC-95 cells originating from primary tumor with no *PPFIA1* amplification, knockdown of liprin-α1 did not have effect on the invasive cell growth. UT-SCC-95 cells have tight cell-cell contacts and adhesion rings, which might explain the lack of effect on cell growth. However, singly embedded liprin-α1 knockdown SCC-25 cells with *PPFIA1* amplification changed the cohesive growth pattern to more efficient invasive growth with less cell-cell contacts and irregular colony morphology in 3D collagen. Liprin-α1 has previously reported functions in cell spreading by stabilization of lamellipodial protrusions, regulation of invadosome dynamics, degradation of extracellular matrix in invasive breast cancer cells, and *in vivo* metastatic cell invasion^11^,^19^,^46^,^47^. Our results support previous findings on the function of liprin-α1 in cell invasive growth and demonstrate morphological changes such as cellular junctions and front-rear cell polarity as well as changes in focal adhesion morphology in invasive cells. Furthermore, in highly invasive breast cancer cells as well as HNSCC cells originating from metastasis or primary persistent as well as primary tumor with *PPFIA1* amplification, liprin-α1 expression correlated with growth properties of the cells. Taken together, these results illustrate that liprin-α1 encompasses oncogenic properties in several actively proliferating and motile HNSCC and breast cancer cells.

To find explanations for differences in liprin-α1 function in different cancer cells, we studied comprehensively the localization of liprin-α1 in different HNSCC and breast cancer cell lines. We found for the first time that in head and neck cancer from primary tumor, liprin-α1 was localized to invadosome containing adhesion ring structures. Interestingly, *PPFIA1* and *CTTN* are located next to each other at the 11q13 amplicon and both the liprin-α1 and cortactin were localized in the same adhesive structures. In contrast, in metastatic or primary persistent HNSCC cell lines as well as in motile and invasive breast cancers, liprin-α1 localized close to focal adhesions near the protruding cell edge, suggesting a function of liprin-α1 in cell migration. In invasive MDA-MB-231 cell line, liprin-α1 knockdown has been shown to affect ECM degradation and cell invasion^11^. We showed that in primary HNSCC cells, liprin-α1 knockdown did not prevent adhesion ring dependent ECM degradation. This was expected since liprin-α1 knockdown did not promote cell invasive growth in non-invasive HNSCC cells. Liprin-α1 depletion resulted in deformation of focal adhesions at the cell periphery in metastatic HNSCC and breast cancer cells, suggesting a role of liprin-α1 in adhesion turnover in these cells. Taken together, our results clearly showed that liprin-α1 localizes to different adhesion related structures depending on the characteristics of the cell line, explaining differences of the function of liprin-α1 in cell adhesion, growth and cell invasion in different tumor cells.

To study the mechanism of liprin-α1 in cell growth and invasion, we carried out gene expression analysis from liprin-α1 knockdown and overexpressing cells. We showed that liprin-α1 knockdown led to vimentin up-regulation in HNSCC cells at gene expression level. Liprin-α1 overexpression, on the other hand, caused the opposite effect on vimentin expression further strengthening the regulative role of liprin-α1 in intermediate filament regulation. In our experiments vimentin upregulation was detected in the insoluble fraction of cells from primary tumor as well as in metastatic cell line lacking adhesion rings, which suggests that vimentin recycling or function was disrupted. Our results demonstrated that in liprin-α1 knockdown cells from metastatic HNSCC *MMP13* and keratins *KRT1, KRT4*, *KRT10* and *KRT13* were significantly underexpressed when vimentin was upregulated. Liprin-α1 knockdown resulted in vimentin overexpression and accumulation in cells which do not express large amounts of vimentin endogenously. Based on our data, we hypothesize that effect of liprin-α1 on adhesion is related to intermediate filament regulation and cells compensate the absence of liprin-α1 and underexpression of keratin intermediate filaments by overexpressing vimentin in HNSCC.

We studied the location of vimentin in HNSCC and breast cancer cells. Vimentin was widely expressed in breast cancer, whereas in metastatic HNSCC it was expressed only in minority of the cells and mostly in cells at the colony edge. In UT-SCC-95 primary HNSCC cells, vimentin was located around adhesion rings, suggesting a function of liprin-α1 and vimentin intermediate filaments in adhesion ring regulation. In MDA-MB-231 breast cancer cells with liprin-α1 knockdown, vimentin cage structure was detected near the nucleus and in metastatic UT-SCC-24B HNSCC cells, vimentin accumulated as aggregates near the nucleus. Furthermore, nuclei were abnormal in shape, or multinucleated and vimentin bridges were formed in these cell lines, demonstrating disturbances in cell division. Previously, dysfunction in vimentin regulation has been demonstrated to lead to vimentin bridges^48^,^49^. Because liprin-α1 silencing and overexpression clearly resulted in changes in vimentin gene and protein expression levels and liprin-α1 knockdown did not affect other epithelial-to-mesenchymal transition related proteins, we suggest that the effects of liprin-α1 silencing on cellular morphology and growth are partly due to the vimentin dysregulation. Overall, our findings show that liprin-α1 is an important regulator of the intermediate filament network in epithelial cancer cells.

Previous studies have revealed several fundamental functions for vimentin in cell adhesion, migration and cellular signaling as well as in cell proliferation and contractibility^44^,^50^,^51^,^52^,^53^. Vimentin regulates β1-integrin recycling^42^ and recently, liprin-α1 was found to be involved in the internalization of integrins in MDA-MB-231 breast cancer cells^43^. To test whether liprin-α1 had any effect on β1-integrin protein levels, we knocked down liprin-α1 in several HNSCC cell lines and discovered that total β1-integrin was accumulating in the soluble fraction of the metastatic cells whereas no effect was found on early or recycling endosomes. Vimentin accumulated only in insoluble fraction of the cells. In non-motile cells, liprin-α1 co-localized with activated β1 integrins and talin in invadosomes containing adhesion rings and it co-immunoprecipitated with talin. Altogether, our results are in line with previous data from invasive breast cancer cells and provide additional evidence on the role of liprin-α1 in β1-integrin signaling also in HNSCC. The function of talin and liprin-α1 in proper dynamics at the cell edge during cell motility and in cell spreading is concentration dependent^19^. Therefore, interactions and balance between liprin-α1 and proteins related to invadosomes and adhesive structures are affecting the invasive behavior of the cells.

Taken together, our results demonstrate that liprin-α1 is a novel regulator of vimentin thereby contributing to cytoskeletal organization as well as to cellular structures related to adhesion in epithelial cancers. Our data implies that liprin-α1 is a potential stabilizer for adhesive structures and a fundamental player in many critical physiological processes in cancer cells.

## Materials and Methods

### Cell lines and reagents

Head and neck squamous cell carcinoma cell lines UT-SCC-19A, UT-SCC-19B, UT-SCC-24A, UT-SCC-24B, UT-SCC-42A, UT-SCC-42B, and UT-SCC-95 were established from clinical squamous cell carcinoma samples of oral tissue by Professor Reidar Grénman (Department of Otorhinolaryngology – Head and Neck Surgery, Turku University Hospital, Finland). CCD-1106 cell line was established from human keratinocytes, SCC-25 from squamous cell carcinoma of oral tongue, MDA-MB-231 from metastatic breast adenocarcinoma and Hs578T from breast carcinoma (ATCC). UT-SCC and MDA-MB-231 cell lines were cultured in Dulbecco’s Modified Eagle’s Medium (DMEM) supplemented with 2 mM L-glutamine, 0,1 mM non-essential amino acids (NEAA), penicillin/streptomycin (100 U/ml) from Lonza and 10% fetal bovine serum (FBS) from Gibco. SCC-25 cells were cultured in 1:1 mixture of Dulbecco’s modified Eagle’s medium and Ham’s F12 medium, containing 1.2 g/L sodium bicarbonate, 2.5 mM L-glutamine, 15 mM HEPES, penicillin/streptomycin (100 U/ml), 0.5 mM sodium pyruvate from Lonza and 10% fetal bovine serum from Gibco. Hs578T cells were cultured in RPMI-1640 medium with 2 mM L-glutamine, 0,1 mM NEAA, penicillin/streptomycin (100 U/ml) from Lonza and 10% FBS from Gibco. CCD-1106 cell line was cultured in SFM keratinocyte medium supplemented with epidermal growth factor (EGF) and bovine pituitary extract (BPE) from Life Technologies as well as penicillin and streptomycin (100 U/ml) from Lonza.

### Constructs

SH002 shScramble (shScr) control and constructs targeting *PPFIA1* and *CTTN* were purchased from TRC.1 library in pLKO.1 vector (Sigma-Aldrich, St. Louis, MO, USA). Five shRNA constructs for both genes were tested and those giving efficient knockdown were selected for further analyses. These constructs were TRCN0000002969 for *PPFIA1* and TRCN0000040274 and TRCN0000040275 for *CTTN*. Open reading frame (ORF) for *PPFIA1* (clone ID 4794300) was ordered from the ORFeome Collection (Open Biosystems, Pittsburgh, PA, USA) and cloned from donor pENTR221 vector into lentiviral destination expression vector pLenti6/V5 DEST (Invitrogen) using Gateway cloning system. Empty pLenti6/V5 DEST vector was used as a control. Gateway cloning was done at the Genome Biology Unit, University of Helsinki, Finland.

### Antibodies

Antibodies for talin (Sigma-Aldrich), vinculin (Sigma-Aldrich), liprin-α1 (Proteintech), cortactin (clone 4F11, Millipore), phalloidin AlexaFluor-594 and AlexaFluor-488 (Life Technologies), activated β1-integrin (12G10, Abcam), total β1-integrin (BD Transduction Laboratories), paxillin (BD Transduction Laboratories), pFAK (BD Transduction Laboratories), vimentin (Sigma-Aldrich), N-cadherin (Abcam), b-actin (Santa Cruz), MT1-MMP (Millipore), Rab11 (BD Transduction Laboratories), EEA1 (BD Transduction Laboratories), PKC_ε_ (BD Transduction Laboratories) and E-cadherin (BD Transduction Laboratories) were used in immunofluorescence and western blotting. Phalloidin-TRITC and collagen type I from rat tail (Sigma-Aldrich) were used in invasion assays. Secondary antibodies goat anti-mouse AlexaFluor-488 and goat anti-rabbit Alexa Fluor-594 (Life Technologies) were used in 1:400 dilution in immunofluorescence. For immunoblotting, secondary antibodies HRP-Goat Anti-Mouse IgG (H + L) and HRP-Goat Anti-Rabbit IgG (H + L) (Life Technologies) were used in 1:10000–20000 dilution.

### Generation of cells with knockdown of *PPFIA1* and *CTTN* by shRNA constructs and overexpression of *PPFIA1* by ORF

Lentiviral particles containing shRNA constructs or ORF for *PPFIA1* were done at the Biomedicum Functional Genomics Unit, University of Helsinki, Finland. Cells were seeded into 12-well plates and incubated overnight. Next day the cells were infected with lentiviral particles containing shRNA or ORF constructs with polybrene (hexadimethrine bromide (8 μg/ml), Sigma-Aldrich), and centrifuged for 30 minutes at 2500 rpm at room temperature. Cells with viruses were incubated 6 hours +37 °C, and after that medium was changed. Transduction was carried out for three days followed by splitting the cells. Selection was done with 1 μg/ml puromycin (Sigma-Aldrich) for shRNA transduced cells and selection was carried out for at least six days. For transduction with overexpression construct selection was done using 2 μg/ml of Blasticidin S HCl (Invitrogen).

### Immunofluoresence

Cells were seeded to glass coverslips and incubated overnight. After cells were attached, they were fixed with 4% paraformaldehyde in phosphate-buffered salin (PBS) for 15 minutes, and washed with PBS. Cells were permeabilized with 0,1% Triton-X for 5 minutes, and incubated with 0,12% glycin in PBS for 10 minutes. Cells were blocked with 3% bovine serum albumin (BSA) for 30 minutes and incubated with primary antibody for 1 hour or overnight in 3% BSA. Cells were washed with PBS and incubated in secondary antibody for 1 hour. Cells were washed with PBS and milli-Q water, and mounted in mounting medium Mowiol, 1,4-diazobicyclo (2,2,2) octane (DABCO) and 4′6-diamidino-2-phenylindole (DAPI) to stain the nucleus.

### Microscopy

Confocal images were collected using Zeiss Meta 780 laser scanning microscope with Zeiss 40x or 63x/1.4 N.A. plan-apochromat oil objective at room temperature. Acquisition was made with Zeiss Zen 2010 Lite program. Images were adjusted with Adobe Photoshop CS6 and Illustrator CS6 software.

### Western blot

Cells were seeded overnight, washed with PBS and lysed to lysis buffer RIPA (Sigma-Aldrich), with protease inhibitors (phosphatase inhibitor cocktail, Roche) and phosphatase inhibitors (complete protease inhibitor cocktail, Roche). Samples were rotated 10 minutes at +4 °C and incubated on ice 30 minutes followed by centrifugation for 13,000 rpm 10 minutes +4 °C. Protein concentrations were measured by RC DC Protein Assay Kit (BioRad). Samples were denatured by boiling them in +95 °C for 5 minutes with added loading buffer (5% β-mercaptoethanol and 2 x Laemmli buffer, BioRad) in ratio 1:1. Ready-made SDS-gels (BioRad) were used, and 5–10 μg protein was loaded into gel. Proteins were blotted from gels to polyvinylidene fluoride (PVDF) membranes (BioRad) using TransferBlot Turbo (BioRad) equipment. Membranes were blocked with 5% milk in tris buffered saline and 0,05% Tween (TBST) for 1 hour, washed with TBST, and incubated with primary antibody for 1 hour to overnight, and washed with TBST. Membranes were incubated with secondary antibody 1 hour, and washed with TBST. Detection reagents were added to membranes (Millipore) and chemiluminescence was detected to X-ray films (Kodak).

### Immunoprecipitation

Cells were counted and plated on cell culture dish and incubated +37 °C overnight. Cells were washed with ice cold PBS and scraped off the dish using a plastic cell scraper. Cells were lysed to lysis buffer containing 20 mM Tris-HCl, 150 mM NaCl, 1 mM sodium orthovanadate, 10 mM NaF, 1 mM EDTA, 1% TX-100 and protease inhibitors. Cell lysates were then pre-cleared by incubating them with immobilized protein A bead (GE Healthcare) slurry for 30 minutes. Pre-cleared cell lysates were incubated in rotation with liprin-α1 antibody for endogenous protein and beads for 2 hours at +4 °C. Tubes were centrifuged, supernatant removed and beads washed in lysis buffer. For immunoblotting, the final supernatant was removed and followed by adding the loading buffer (5% β-mercaptoethanol and 2 × Laemmli buffer, BioRad). Samples were boiled in +95 °C for 5 minutes to denature the protein and separate it from the beads, samples were centrifugated and supernatant collected, after which western blot was performed.

### Colony growth formation assay

0,7% bottom agar layer was prepared day before plating the cells onto 6-well plate and incubated in +37 °C overnight. Next day cells were calculated (5000 cells/ml) and mixed with medium and 0,35% top agar and plated on 6-well plates on top of bottom agar. Plates were incubated on +37 °C for three weeks. Colonies were stained with 0,005% crystal violet/methanol, and incubated overnight +4 °C. Colonies were washed with Milli-Q water several times. Colonies were imaged with Leica MZ FL III stereomicroscope. Camera was The Soft ImagingSystems View Fire Wire camera and software Soft ImagingSystems analySIS. Quantification and calculations were made with Adobe Photoshop CS6 software.

### Cell growth assay

Cells were trypsinized and counted, and seeded onto 96-well plate. Cells were incubated for 2–6 days, and thiazolyl blue tetrazolium bromide (MTT, 0,5 mg/ml) reagent was added to cells to assess cell viability and growth. MTT was incubated with cells 2 hour after which cells were lysed to lysis buffer (10% SDS, 10 nM HCl), and incubated +37 °C overnight, after which absorbance was measured with Victor plate reader at wavelength at 540 nm.

### Gelatin degradation assay

Coverslips were coated with poly-L-lysine (50 μg/ml, Sigma-Aldrich), and washed with PBS. Coverslips were fixed with 0,5% glutaraldehyde (Sigma-Aldrich), and washed with PBS. Coverslips were coated with 1 mg/ml Oregon green gelatin mixed with 2 mg/ml denatured collagen I, and washed with PBS. Coverslips were incubated with 5 mg/ml sodium borohydride (Sigma-Aldrich), and washed with PBS. Coverslips were incubated in complete medium overnight. Cells were counted and seeded to coverslips. Cells were incubated in complete medium, and fixed with 4% PFA.

### Quantification of focal adhesions and extracellular matrix degradation

Focal adhesions and extracellular matrix degradation were quantified by ImageJ software. After thresholding the background, vinculin positive focal adhesions were quantified by function analyzing particles. Average size or shape of focal adhesions were quantified by analyzing three times 30 cells from different experiments. Focal adhesions were defined by intensity threshold, and images were inverted to black and white. Area of focal adhesions were set from five to infinity and circularity was set from 0 to 1 of the total area. To quantify particles with elongated shape, circularity was set from 0 to 0,5. For quantification of extracellular matrix degradation, background was first defined by intensity threshold and degradation area was set from one to infinity and circularity from 0 to 1. For quantification of extracellular matrix >50 cells/experiment were analyzed. Error bars were calculated from standard deviations of three experiments. Unpaired student’s *t*-test was used for statistical analysis and result was regarded significant if P < 0.05. Confocal images were collected using Zeiss Meta 780 laser scanning microscope with Zeiss 40x/1.4 N.A. plan-apochromat oil objective at room temperature.

### Microarray analysis

RNA from UT-SCC-95 and UT-SCC-24B cell lines was extracted using miRNeasy Mini kit from Qiagen according to manufacturer’s protocol. The RNA was analyzed for integrity and quality on Agilent Bioanalyzer 2100. Two different arrays were used. UT-SCC-95 was hybridized into Affymetrix Human Exon 1.0 arrays. Starting amount of total RNA was 100 ng and 15 μg of cRNA and 5,5 μg of sscDNA were used for labeling. The labeling and hybridization were done according to the manufacturer’s instructions. Protocols used for arrays were the Ambion WT Expression Kit for Affymetrix GeneChip Whole Transcript (WT) Expression Arrays (Part 4425209 Rev. C) and GeneChip WT Terminal Labeling and Hybridization User Manual for use with the Ambion WT Expression Kit (P/N 702808 Rev. 4). One-hundred nanograms of total RNA extracted from UT-SCC-24B cell line was hybridized into Affymetrix Human Gene 2.0 ST arrays, and 15 μg of cRNA and 5,5 μg sscDNA were used for labeling. The labeling and hybridization were done according to the manufacturer’s instructions. Protocols and reagents for Human Gene 2.0 ST arrays were GeneChip® WT Plus Reagent Kit, Manual Target Preparation for GeneChip® Whole Transcript Expression Arrays (P/N 703174 Rev.2), Affymetrix GeneChip® Expression Wash, Stain and Scan User Manual For Cartridge Arrays (P/N 702731 Rev. 3, Affymetrix). The microarray data discussed in this publication have been deposited to NCBI’s Gene Expression Omnibus and are accessible through GEO Series accession number GSE75756 (http://www.ncbi.nlm.nih.gov/geo/query/acc.cgi?&acc=GSE75756).

### 3D type I collagen growth and invasion assays

Type I collagen (2,2 mg/ml) was prepared; invasion and growth were assessed essentially as described^54^. For invasive growth assay, single cell suspension with 3000 cells was mixed in collagen and cultured for 5 (Fig. 1) or 9 (Fig. 4) days. Cultures were fixed and the cells were visualized by staining with phalloidin for filamentous actin and DAPI for nuclei.

For quantification of invasive growth, the area of invading and non-invading cell colonies were measured by outlining the colony boundary and calculating the area using Image J software. In addition, the number of nuclei were counted from each cell colony from random epifluorescence images of at least three gels. The invasive growth, expressed as the ratio of cell colony area/number of nuclei, represents the level of cell colony branching in 3D collagen. Alternatively, the number of colonies displaying invasive growth as cellular sprouts was counted and invasive growth was expressed as the ratio of invasive colonies/total colonies (Fig. 4A,B). The numerical values represent mean +/− SEM in invasive growth assays. Statistical significance was determined using the unpaired Student’s *t*- test.

### Transwell migration assay

Transwell cell migration assay was performed using 24-well plates (BD Biosciences) with cell culture inserts (8 μm pore size in the membrane, BD Biosciences). For analysis of cell migration, the upper surface of the membrane was coated with Matrigel without growth factors diluted in cold serum-free DMEM at the concentration 200 μg/ml and incubated at 37 °C for 1 h to allow the Matrigel to solidify. Cells were washed with PBS, resuspended in the medium with 10% FBS and incubated at 37 °C for 2 h. After incubation cells were washed three times with PBS and resuspended in serum-free medium supplemented with 0.1% albumin solution (BSA, Sigma-Aldrich). Cell suspension (8 × 10^4^ cells per ml, 250 μl) was added to the upper chamber, and complete medium (750 μl) with 10% FBS was added to the bottom wells of the chambers. The cells that had migrated to the lower surface of the membranes were fixed with methanol for 20 min, stained with Hoechst and quantified using Cellomics ArrayScan 4.5 (Thermo Scientific). The upper surface of the chambers were cleaned using cotton swabs. Images of 30 fields were captured from each membrane and the number of cells migrating through the insert was counted. The average of duplicate assays for each experimental condition was used, and experiment was repeated three times. The error bars were calculated from standard deviations from three different experiments.

### Spheroid formation assay

Cells were counted and 10000 cells/well were plated to low adhesion plates (Corning), where cells where allowed to form spheroid structures for four days. Spheroids were imaged with phase contrast microscope Motic and software Motic. Images were processed with Adobe Photoshop CS6 and Adobe Illustrator CS6.

### Soluble and insoluble fractioning

Cells were incubated at +4 °C for 30 minutes with phosphobuffer (NaCl 150 mM, Hepes 20 mM pH 7,6, 1% Igepal, 10% glycerol) containing phosphatase inhibitors (phosphatase inhibitor cocktail, Roche), and protease inhibitors (complete protease inhibitor cocktail, Roche) followed by scraping them off from cell culture flasks. Samples were centrifuged at 20,000 g at +4 °C for 20 minutes. The supernatant (soluble fraction) was collected while the pellet (insoluble fraction) was washed three times with 2 mM EDTA in PBS, resuspended in Triton buffer (PBS, 1% SDS, 0,1% Triton), and then boiled for 10 min, and pipetted through a needle several times^55^. Soluble and insoluble fraction were separated by SDS page, transferred to PVDF-membrane and probed with the antibodies, and detected by chemiluminescence.

## Additional Information

**How to cite this article**: Pehkonen, H. *et al.* Liprin-α1 is a regulator of vimentin intermediate filament network in the cancer cell adhesion machinery. *Sci. Rep.* **6**, 24486; doi: 10.1038/srep24486 (2016).

## Supplementary Material

Supplementary Information

## Figures and Tables

**Table 1:** Characteristics of the cell lines used in the study.

Cell line	Description of the cell line	Location	P/M	11q13+
MDA-MB-231	breast adenocarcinoma	breast	M	no
Hs578T	breast carcinoma	breast	P	no
SCC-25	head and neck squamous cell carcinoma	tongue	P	yes
UT-SCC-19A	head and neck squamous cell carcinoma	larynx	P	no
UT-SCC-19B	head and neck squamous cell carcinoma	larynx	P (per)	yes
UT-SCC-24A	head and neck squamous cell carcinoma	tongue	P	yes
UT-SCC-24B	head and neck squamous cell carcinoma	neck	M (per)	yes
UT-SCC-42A	head and neck squamous cell carcinoma	tongue	P	no
UT-SCC-42B	head and neck squamous cell carcinoma	neck	M	no
UT-SCC-95	head and neck squamous cell carcinoma	tongue	P	no

Abbreviations: P = primary tumor, M = metastasis, per = persistent disease, 11q13+ = cases with 11q13 gain or amplification.

**Table 2:** Differential expression of genes related to intermediate filaments and extracellular matrix in UT-SCC-24B cells with *PPFIA1* knockdown.

Gene	Description	FC log 2	p-value
VIM	vimentin	2,5	0,00105
KRT4	keratin 4	−1,9	0,000787
KRT10	keratin 10	−2,47	0,000361
MMP13	matrix metallopeptidase 13 (collagenase 3)	−2,3	0,000361
PPFIA1	protein tyrosine phosphatase, receptor type, f polypeptide (PTPRF), interacting protein (liprin), alpha 1	−0,98	0,01315
KRT13	keratin 13	−1,59	0,007413
KRT1	keratin 1	−1,29	0,012416

Cells with liprin-α1 knockdown were compared to control cells containing non-target shRNA. Fold change (FC) is in log 2 scale and it has been calculated from three replicates.

**Table 3:** Differential expression of genes related to intermediate filaments and extracellular matrix in UT-SCC-95 cells with ectopic expression of *PPFIA1*.

Gene	Description	FC log 2 replicate1	FC log 2 replicate2	Average FC log 2
PPFIA1	protein tyrosine phosphatase, receptor type, f polypeptide (PTPRF), interacting protein (liprin), alpha 1	1,89	2,42	2,16
VIM	vimentin	−2,04	−2,07	−2,05
MMP13	matrix metallopeptidase 13 (collagenase 3)	1,18	1,06	1,12

Cells with liprin-α1 expression were compared to corresponding control cells containing empty vector. Fold change (FC) is in log 2 scale and the values are shown separately from two replicates.
