# Supplementary material for: Liprin-α1 is a regulator of vimentin intermediate filament network in the cancer cell adhesion machinery
Source: Sci Rep. 2016 Apr 14;6:24486. doi: 10.1038/srep24486 (PMC4830931; doi:10.1038/srep24486)
Supplement: Supplementary Information [file srep24486-s1.pdf]

Supplementary Data

Liprin-α1 is a regulator of vimentin intermediate filament network in the cancer cell adhesion machinery

Henna Pehkonen[1], Pernilla von Nandelstadh[1], Piia-Riitta Karhemo[1], Tatiana Lepikhova[1], Reidar Grenman[2], Kaisa Lehti[1,3,4], Outi Monni[1]*

[1]Research Programs Unit, Genome-Scale Biology Research Program and Institute of Biomedicine, Medical Biochemistry and Developmental Biology, 00014 University of Helsinki, Finland, [2]Department of Otorhinolaryngology, Head and Neck Surgery, Turku University and Turku University Hospital, Finland; [3]Department of Microbiology, Tumor and Cell Biology, Karolinska Institutet, Stockholm, Sweden; [4]K. Albin Johansson Senior Researcher, Finnish Cancer Institute, Finland

*Corresponding author

Outi Monni, PhD

Research Programs Unit,

Genome-Scale Biology Research Program

and Institute of Biomedicine

Medical Biochemistry and Developmental Biology

Biomedicum Helsinki 1, Rm B326a

POBox 63 (Haartmaninkatu 8)

FIN-00014 University of Helsinki

FINLAND

Tel: +358-40-7639302

E-mail: outi.monni@helsinki.fi

A.

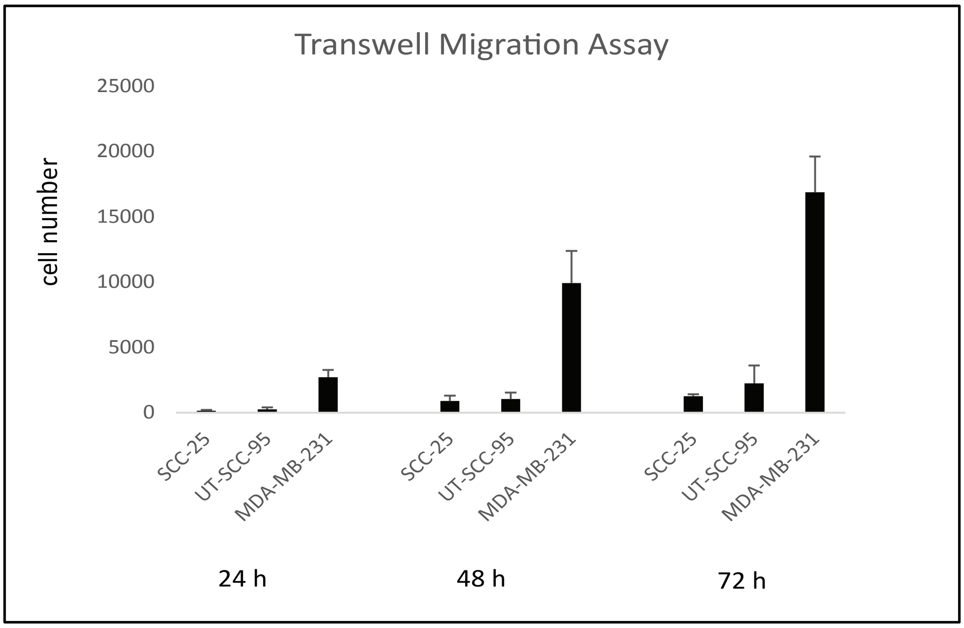

B.

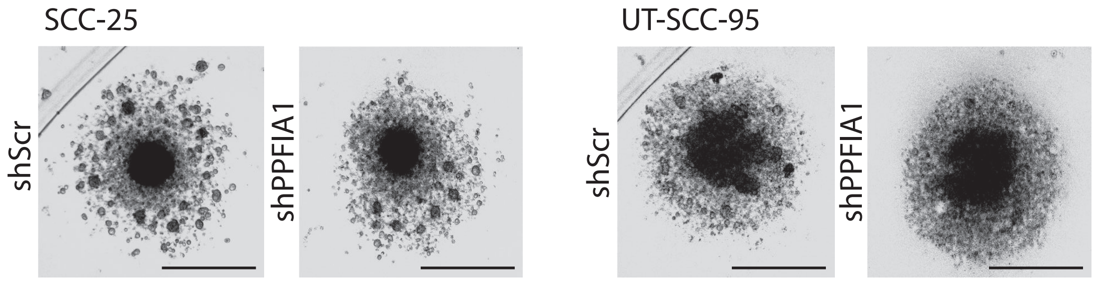

Supplementary Figure 1. A: Comparison of HNSCC cell lines SCC-25 and UT-SCC-95 from primary tumor and the invasive breast cancer cell line MDA-MB-231 in the transwell migration assay through matrigel. Experiments were done three times in three different time points, and error bars indicate standard deviations. B: Spheroid formation assay of HNSCC cell lines with control shScr and shPPFIA1 cells. The scale bar is 0,6 mm.

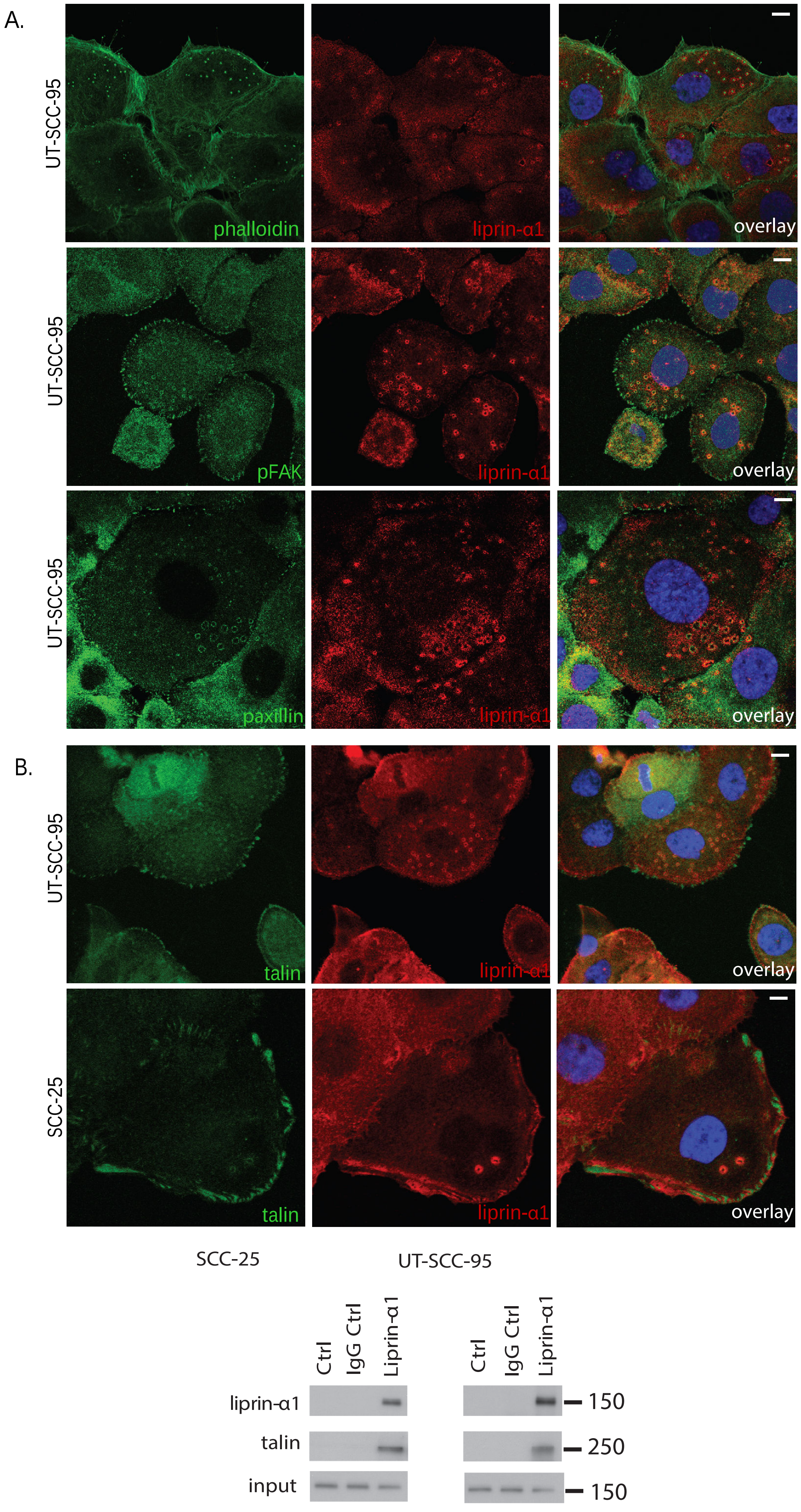

Supplementary Figure 2. A: Localization of actin (phalloidin), pFAK and paxillin in adhesion rings together with liprin-α1 in the UT-SCC-95 cell line. B: Talin and liprin-α1 localization in adhesion rings in UT-SCC-95 and SCC-25 HNSCC cell lines. Co-immunoprecipitation of liprin-α1 with talin in SCC-25 and UT-SCC-95 head and neck cancer cells. Two control samples were used: one with no antibody and the other with non-specific IgG antibody. Immunoprecipitation was performed using liprin-α1 antibody. The scalebar is 10μm.

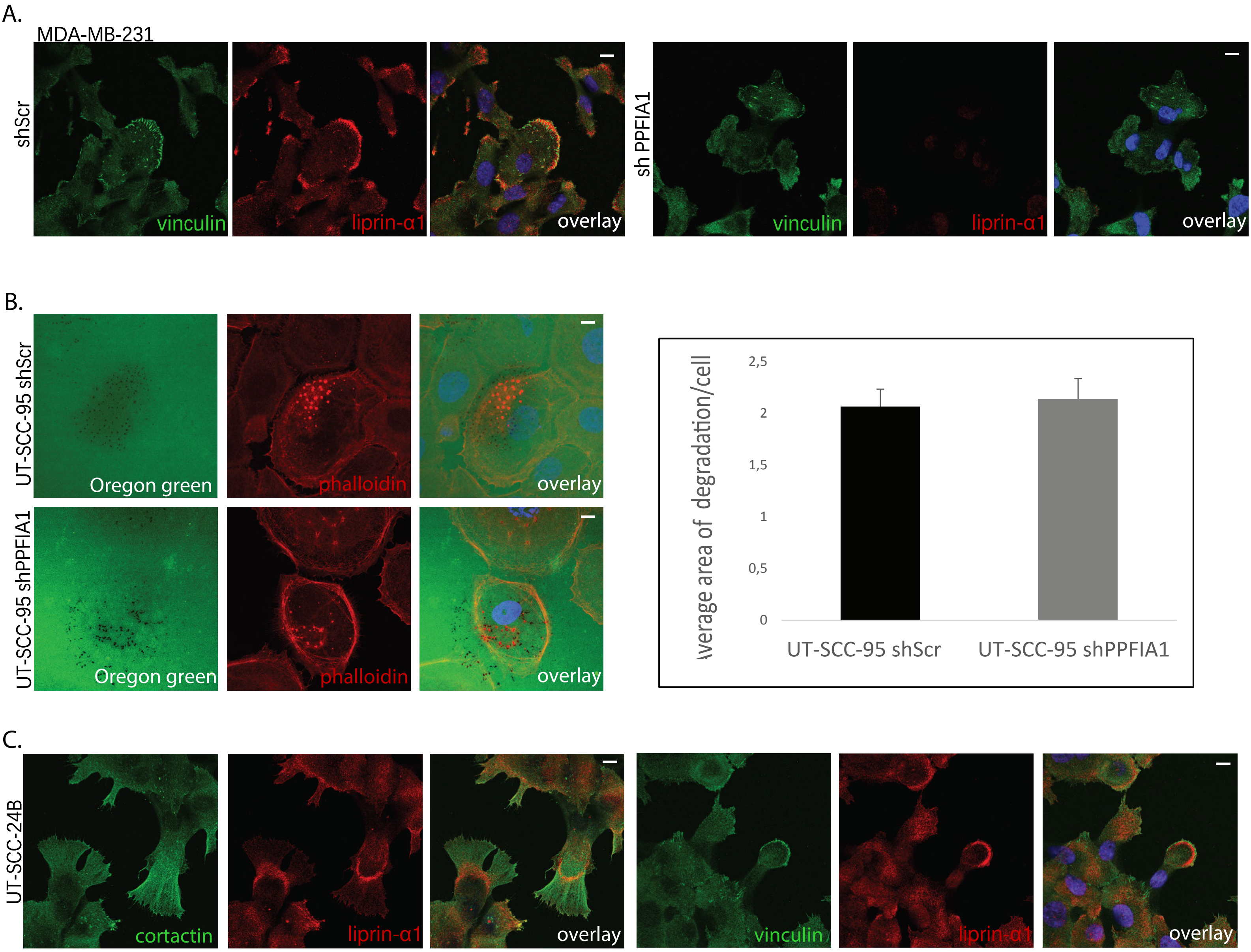

Supplementary Figure 3. A: Liprin-α1 localized after leading edge with vinculin in control MDA-MB-231 cells, whereas in liprin-α1 knockdown cells vinculin showed more dispersed staining. B: In adhesion ring dependent extracellular matrix degradation from primary HNSCC cell line liprin-α1 did not inhibit degradation of extracellular matrix. Extracellular matrix degradation was measured by gelatin degradation assay for UT-SCC-95 HNSCC cell line. Quantification of extracellular matrix degradation was carried out from three experiments with >50/cells/experiment. Incubation time for quantification was 24 hours. C: Localization of liprin-α1 behind the leading edge in metastatic HNSCC cell line. Liprin-α1 did not directly co-localize with cortactin or vinculin. The scale bar is 10 µm.

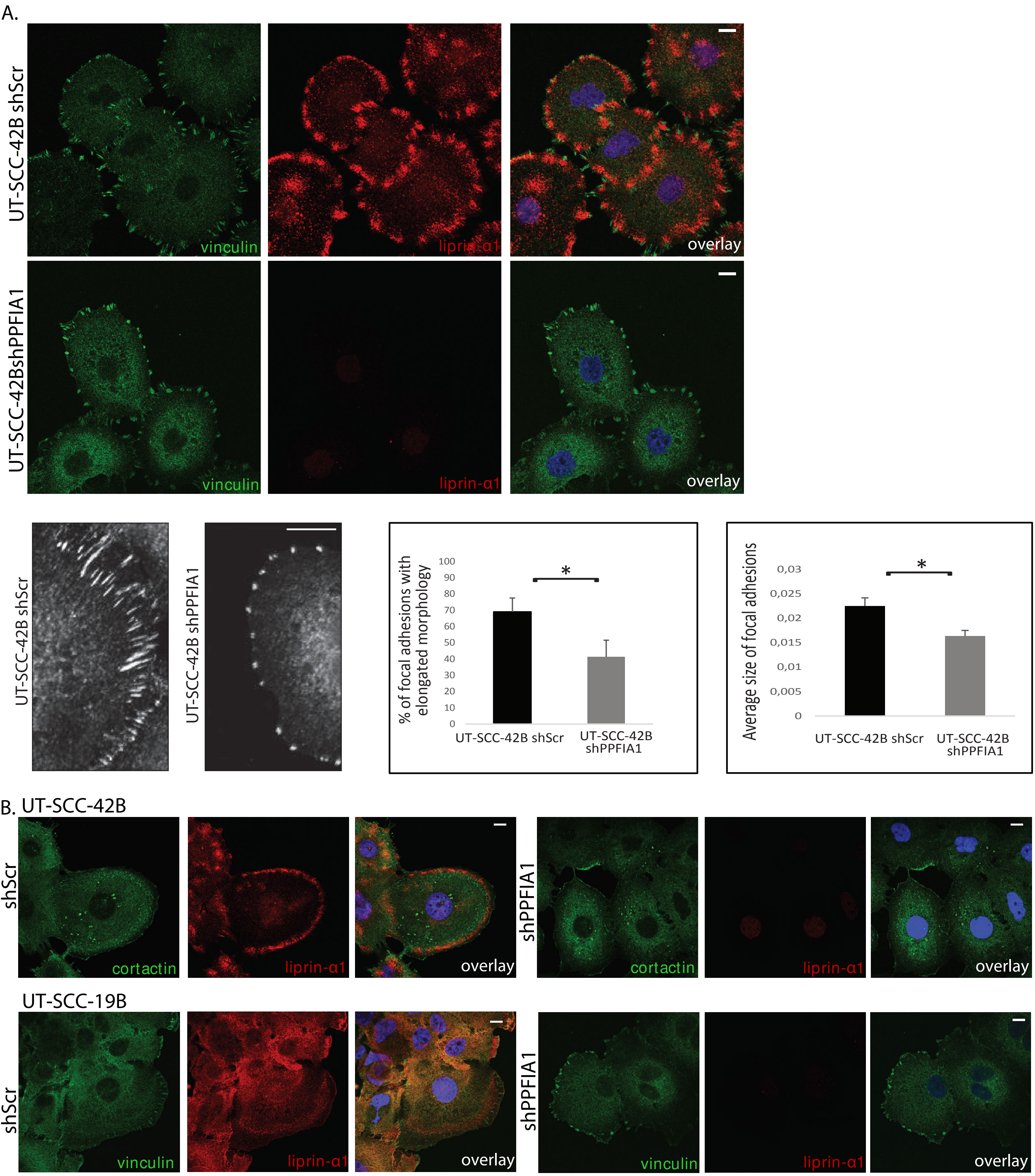

Supplementary Figure 4. A: Liprin-α1 knockdown influenced on the shape and average size of focal adhesions as shown by vinculin staining in UT-SCC-42B and quantification of focal adhesions. Experiments were done three times with 30cells/experiment. Error bars indicate standard deviations.B: Liprin-α1knockdown did not prevent cortactin positive structures in the UT-SCC-42 cell line. Liprin-α1 knockdown resulted in changes in shape and size of focal adhesions in the UT-SCC-19B cell line. The scale bar is 10μm.

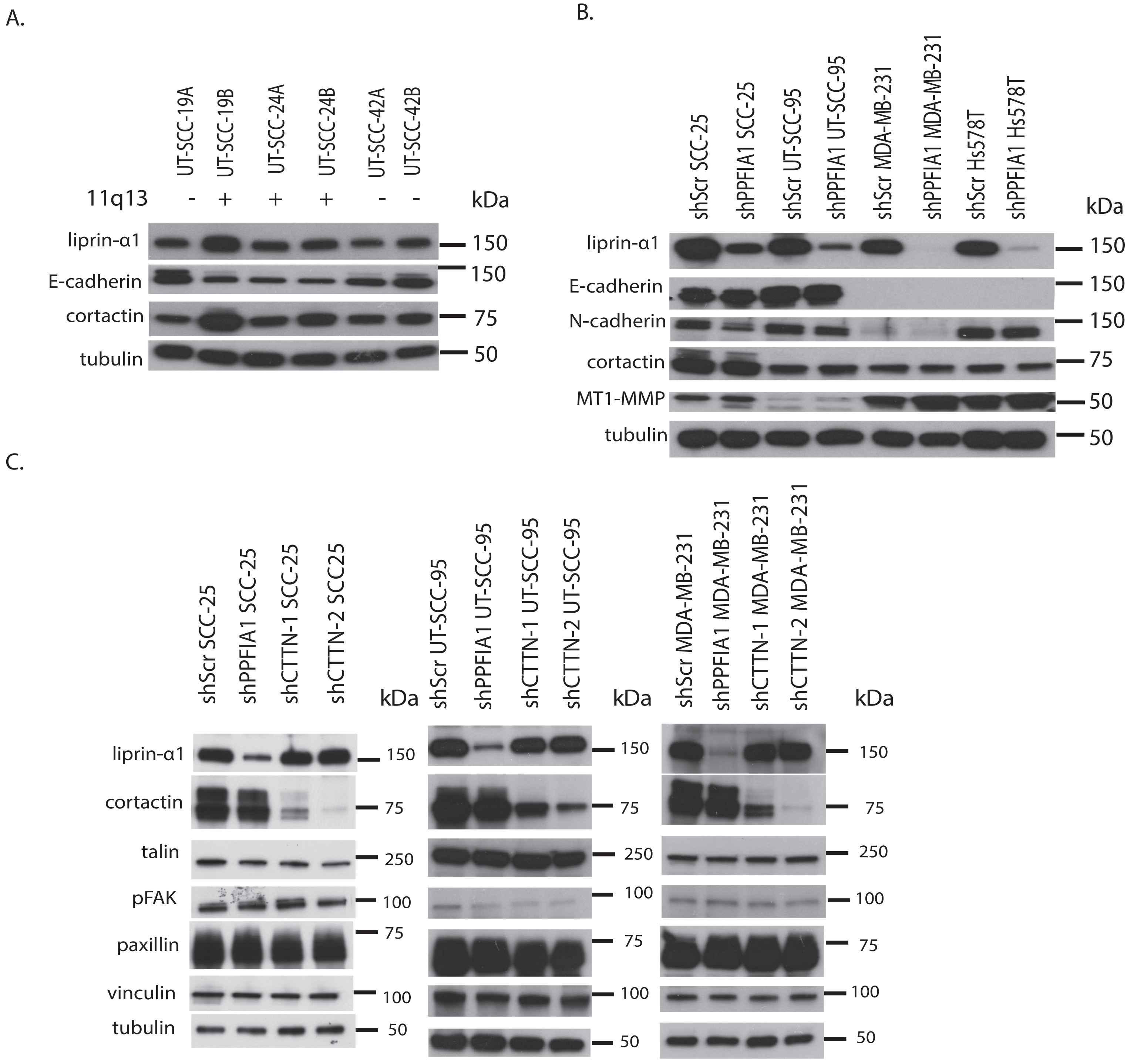

Supplementary Figure 5A:Western blot showing liprin-α1, cortactin and E-cadherin levels in primary HNSCC and their corresponding metastatic or primary persistent cell lines. UT-SCC-19B has high-level amplification of 11q13 whereas UT-SCC-24A and B has low-level amplification spanning a large chromosomal region at the 11q. B: Cadherins, cortactin and MT1-MMP levels in HNSCC and breast control (shScr) and liprin-α1 knockdown (shPPFIA1) cell lines. C: Immunoblot showing no significant difference in protein expression of different focal adhesion proteins in HNSCC and breast cancer cell lines after knockdown of liprin-α1 and cortactin.
